# Acquisition of Demonstratives in English and Spanish

**DOI:** 10.3389/fpsyg.2020.01778

**Published:** 2020-07-24

**Authors:** Patricia González-Peña, Martin J. Doherty, Pedro Guijarro-Fuentes

**Affiliations:** ^1^School of Psychology, Faculty of Social Sciences, University of East Anglia, Norwich, United Kingdom; ^2^Departament de Filologia Espanyola, Moderna i Clàssica, Universidad de las Islas Baleares, Palma, Spain

**Keywords:** English, Spanish, deixis, spatial demonstratives, language acquisition, corpus linguistics, CDI

## Abstract

The present work re-evaluates the long-standing claim that demonstratives are among infants’ earliest and most common words. Although demonstratives are deictic words important for joint attention, deictic gestures and non-word vocalizations could serve this function in early language development; the role of demonstratives may have been overestimated. Using extensive data from the CHILDES corpora (Study 1, *N* = 66, 265 transcripts) and McArthur-Bates CDI database (Study 2, *N* = 950), the language production of 18- to 24-month-old Spanish- and English-speaking children was analyzed to determine the age and order of acquisition, and frequency of demonstratives. Results indicate that demonstratives do not typically appear before the 50th word and only become frequent from the two-word utterance stage. Corpus data show few differences between Spanish and English, whereas parental report data suggest much later acquisition for demonstratives in English. These findings expand our knowledge of the foundations of deictic communication, and of the methodological challenges of assessing early production of function words.

## Acquisition of Demonstratives in English and Spanish

Infants communicate about objects and locations in space early in development. By interacting with their caregivers in relation to an object, they are engaging in *deictic communication.* This happens by 12 months, before children have learnt their first words, with the onset of pointing (Tomasello et al., 2007). Pointing is a deictic gesture, and is crucial in language acquisition as it supports word learning and facilitates the transition to two-word utterances (Iverson and Goldin-Meadow, 2005). Demonstrative words (*here*, *there*, *this*, and *that*) are deictic terms. They function to establish joint attention, and often appear in conjunction with pointing (Diessel, 2006; Todisco et al., in press). Given the importance of deictic pointing in language acquisition, it is plausible that demonstratives also have a central role, and therefore would be some of the first and most frequent words of infants - this assumption has been conventional in the literature (Clark, 1978; Clark and Sengul, 1978). Clark claimed that demonstratives are typically acquired among the first 10 words, and always among the first 50. Her claim was based on observational studies with English speaking American children (Nelson, 1973; Braine and Bowerman, 1976) and single-case diaries of other languages. However, no systematic empirical work has addressed this issue.

Given the recent growth of child language databases and the emergence of tools to process them, it now seems appropriated to re-evaluate the claim that demonstratives appear at the start of language development, and are thus foundational to deictic communication and word learning. Several works on child early speech challenge the claim of an early acquisition of demonstratives. Caselli et al. (1995) described the language acquisition of English and Italian speakers based on parental report with the MacArthur-Bates Communicative Development Inventory (CDI) on over 800 children, and did not find any demonstratives among the 50 words first produced in either language. These data are striking but inconclusive, since the sensitivity of parental report to detect function words in child vocabulary is as yet unclear (Salerni et al., 2007). Rodrigo et al. (2004) observed deictic communication in child-mother dyads. They found deictic words to be rare before the age of two and more frequent afterward, whereas younger infants established joint attention often by using a non-word vocalizations in combination with pointing. In line with this, Capirci et al. (1996) found a small proportion of deictic words in 16- and 20-month-old Italian infants, and a greater proportion of deictic gestures (in combination or not with a content word).

This evidence challenges the idea that demonstratives are essential words in early child speech. It instead suggests that deixis in early stages of language acquisition could rely on gestures, or verbal expressions other than demonstratives.

The aim of this work is to test the claim of an early acquisition of demonstratives to assess the role of these words in language development and deictic communication in infancy. To that aim, we look at child productive speech between 18 and 24 months, which encompasses the typical onset of expressive language and development toward two- or multi-word utterances. We compare demonstrative acquisition in two languages, English and Spanish, chosen because of the differential characteristics of their demonstrative systems (greater syllabic and morphological complexity in Spanish) and because both languages have a large amount of data available as open source for study. Data are obtained from two large repositories of child language acquisition: the CHILDES corpus, comprising transcripts of child spontaneous speech, and the MacArthur-Bates CDI Wordbank, comprising data from parental surveys. A secondary aim is to describe the use of demonstratives in English and Spanish in infant speech and parent-directed speech.

Demonstratives in English are the words *this* and *that* (and their plural forms *these* and *those*) and the locative adverbs *here* and *there*. *This* and *that* can function as pronouns (e.g., “what is *that*?”) or determiners (e.g., “*that* book on the right”). Most authors include locative adverbs in the category of demonstratives (Diessel, 2006), although their functions differ slightly; locative adverbs specify a place, whereas determiners and pronouns refer to an object, and are often not used with the aim of disambiguating object position. Spanish demonstratives have three terms instead of two, for proximal, medial and far distance, and vary not only in number but in grammatical gender. See Table 1 for a full list of Spanish demonstratives. We will compare data from determiners/pronouns with data from the locative adverbs, and ask whether they might have different roles in child speech and be acquired at different times. To preview the results, locatives appear to be acquired earlier, particularly in English, and unlike determiners/pronouns, they do not correlate with language development, measured by mean length of utterance (MLU). Thus, determiner/pronouns and locatives may have different roles.

**TABLE 1:** Demonstrative words in Spanish.

		**Proximal**		**Medial**		**Distal**	
		**Det/pronoun**	**Locative**	**Det/pronoun**	**Locative**	**Det/pronoun**	**Locative**
Singular	Male	este		ese		aquel	
	Female	esta		esa		aquella	
	Neutral	esto	aquí	eso	ahí	aquello	allí
Plural	Male	estos	acá	esos		aquellos	allá
	Female	estas		esas		aquellas	

*Spanish locative adverbs *aquí* and *acá*, and *allí* and *allá* will be treated as synonymous in our work.*

### Sources of Child Speech Data

The CHILDES project is a collection of corpora that feature transcripts of first language acquisition (MacWhinney, 2000). The earliest transcripts date back to the 1973, and it has grown greatly since. The *childesr* package for the statistical software R now allows extracting data from all selected transcripts simultaneously. The MacArthur-Bates CDI (Fenson et al., 2007) is a family of parent inventories that collect data of child expressive and receptive vocabulary and gestures in multiple languages. It has been extensively used as a measure of language development for over 20 years. Since 2017, data are available to use in a structured database called Wordbank, that features data from more than 75000 children^1^ (Frank et al., 2016).

As methods for the study of child language acquisition, the analysis of spontaneous speech and parent report have different strengths and potential biases. The advantage of CHILDES data is that they feature naturalistic language production, including parent child-directed speech. However, they do not contain the child’s total vocabulary size, and the words in a transcript might be task biased, and not fully representative of child speech in other contexts. The CDI’s main strengths are very large sample sizes and that it applies the same items to all children. Abundant studies support the CDI as a reliable and valid measure of child language development (Dale et al., 1989; Feldman et al., 2005) with high predictive validity even several years later (Can et al., 2013). However, CDI data could underestimate function words in children’s vocabulary, as opposed to child corpora, where they might be overrepresented (Salerni et al., 2007). Demonstratives are generally studied within the category of function words in the literature in language acquisition, together with words such as articles, prepositions, and conjunctions (Caselli et al., 1995; Salerni et al., 2007). Moreover, it has been suggested that parents from low socioeconomic status background (SES) could be less accurate at reporting their child’s vocabulary in inventories. Higher CDI scores have been reported for low SES children relative to high SES children, whereas the literature has consistently reported a disadvantage in language acquisition for children from low SES backgrounds (Reznick, 1990; Fenson et al., 1994). In the case of function words, the demographic differences in parental report might be higher, because these words might be harder to detect (Fenson et al., 1994). Thus, it has been suggested that neither corpus data nor parent report are ideal methods on their own to estimate the frequency of a particular word type in child speech, and using both in combination has been recommended (Pine et al., 1996; Salerni et al., 2007).

To sum up, the principal aim of this work is to study the emergence and frequency of demonstratives in early child speech in order to re-evaluate our knowledge about the function of demonstrative words in early stages of language acquisition. An early acquisition of demonstratives (among the first 10 or 50 words as suggested by Clark) and high frequency would indicate an essential role of this word class for language acquisition and communication. Contrarily, a later acquisition or marked differences between-languages would support the hypothesis that demonstratives are just one of the possible forms of deixis, and not essential to language acquisition. Specifically, the acquisition of the first demonstrative words will be examined in relation to chronological age, mean length of utterance (MLU, in corpus data) and estimated vocabulary size (CDI data). Study 1 will examine the data from spontaneous speech and Study 2 from parent report.

Additionally, we compare the use of determiners/pronouns with that of locatives. Subtle differences between the two types of term may affect their developmental trajectory. We also compare parent and child use of demonstratives in the same conversation to examine whether parents tend to adopt the demonstratives used by the child regardless of their own perspective.

To preview the results, we find that demonstrative words do not typically appear among the first 50 words, and are more frequent in child’s speech toward the age of two years and in two- and multi-word utterances than in the earliest stages of language acquisition. We find cross-linguistic differences, namely late acquisition of demonstratives in English with respect to Spanish. However, these differences are evident only in parental report data. The discussion will cover the implications for deictic communication and methodological considerations regarding the study of function words in child speech.

## Study 1: CHILDES Corpora

Study 1 investigates the acquisition and use of demonstrative words using data from spontaneous speech.

### Method

#### Origin of the Data

Data come from monolingual children aged 18 to 24 months from the European Spanish and British English corpora in CHILDES (MacWhinney, 2000). All transcripts that fit these criteria and included an interaction with the mother or father were selected. Seven Spanish corpora (Linaza, Vila, SerraSole, Aguirre, OreaPine, Nieva, and Ornat) and six British English corpora (Forrester, Wells, Manchester, Lara, Howe, and Cruttenden) were included. The British sample comprised 173 transcripts from 59 children, and the Spanish sample 92 transcripts from seven children (see descriptives in Table 2). The number of transcripts per child ranged from one to 39, and they will be analyzed as independent data. Transcripts contained between 9 and 840 target-child utterances (*M* = 240, *SD* = 156); *t*-tests confirmed that there are no significant differences between languages in the number of child utterances by transcript for each of the age groups 18–20 months, 21–22 months, and 23–24 months (all *p’*s > 0.3).

**TABLE 2:** Mean length utterance (MLU) and number of word types (number of different words) of the transcripts used, displayed by age and language.

	**Spanish**				**English**			
**Age (months)**	**N of transcripts**	**N of children**	**MLU Mean (*SD*)**	**Word types Mean (*SD*)**	**N of transcripts**	**N of children**	**MLU Mean (*SD*)**	**Word types Mean (*SD*)**
18	2	1	0.97	23	20	19	1.13	40.75
			(0.07)	(15.56)			(0.2)	(27.29)
19	18	5	1.65	43.5	18	18	1.19	78.11
			(0.5)	(17.47)			(0.21)	(47.52)
20	8	3	1.41	132	13	11	1.48	74.08
			(0.18)	(45.68)			(0.36)	(56.24)
21	22	6	1.65	79.77	31	23	1.58	73.39
			(0.41)	(44.43)			(0.38)	(50.47)
22	20	5	1.63	121.5	16	6	1.65	115.13
			(0.36)	(63.63)			(0.26)	(23.06)
23	22	5	1.79	142.45	75	24	1.66	110.71
			(0.39)	(65.91)			(0.41)	(41.53)
Total	92	7	1.64	100.04	173	59	1.54	90.2
			(0.41)	(63.24)			(0.39)	(48.69)

*MLU was calculated on the number of words instead of morphemes, because the number of morphemes was not available for all transcripts. Therefore, unintelligible vocalizations (in the transcripts, xxx) were computed as words, and contracted forms (*I’m, what’s)* were computed as one word.*

Parent data were obtained in most cases from maternal transcripts, because they were much more frequent than paternal transcripts and generally had more utterances. Paternal transcripts were used when maternal transcripts were not available. In the case of one child of the Spanish corpus (12 transcripts), the father was selected for all instances, because the mother had few utterances and was absent in three of them.

#### Data Processing and Analysis

Data were extracted and processed in R (R Core Team, 2018) in December 2019 using the R package *childesr* (Braginsky et al., 2019). The number of occurrences of each demonstrative word for parent and child was computed. In Spanish we extracted proximal, medial and distal pronouns/determiners and locative adverbs (*este, ese, aquel^2^* including gender and number inflections and *aquí, ahí* and *allí*, see Table 1) and English proximal and distal terms (*this, that, these, those, here* and *there*). In English, demonstratives also have non-deictic uses, such as *there is/are* to indicate existence or in fixed expressions such as *there you go*, and the conjunction *that* (as in *the lady that we met today*). This is not the case for Spanish. We were concerned about the possibility of children using these words non-deictically prior to the acquisition of proper demonstrative use in English. Thus, we checked manually the transcripts of the 10 children from the English corpus who produced only *that* or *there*, which could indicate this non-deictic usage (e.g., in the fixed expression *there you go)*. In all cases we found they apparently functioned as demonstrative words^3^.

All statistical analyses were performed on the raw frequencies. Due to differences in sample size between languages and the violation of the normality and homoscedasticity assumptions, non-parametric tests were used: Chi-squared tests (χ^2^) were used for dichotomous variables and Mann-Whitney *U* Tests for continuous variables with Bonferroni adjusted alpha levels for multiple comparisons. The correlational analysis was performed with bootstrap.

### Results

First, we describe children’s acquisition of demonstrative words with respect to age and MLU, and which demonstrative terms appear in infancy. We then examine whether demonstratives are among children’s most frequent words in our sample. Next, we look at the frequency of use of demonstratives per thousand words through development and in comparison with adult use. Finally, we test whether parents and children tend to use the same or opposite demonstrative terms within a conversation. The acquisition of the correct gender and number demonstrative forms as well as the distance contrast conveyed with demonstratives are not within the scope of this work.

#### Emergence of Demonstratives in Child Speech

**FIGURE 1:**
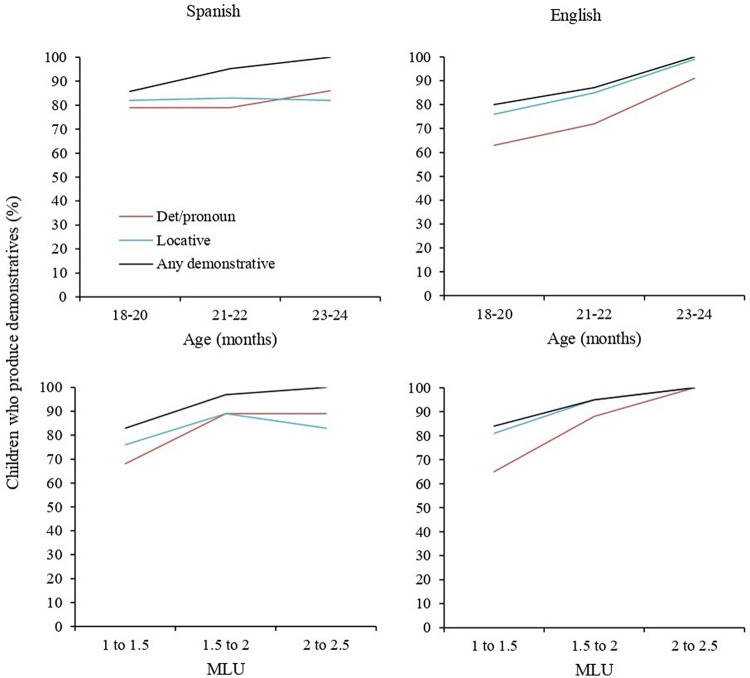
Children who produce at least one demonstrative word in CHILDES corpora, by language, above by Age and below by MLU (%).

We first looked at the percentage of children who used at least one demonstrative word by age and by MLU (see Figure 1). A minimum of 60% of children used at least one demonstrative word at any age and MLU point for either language. Over 80% of children used demonstratives from the single word stage (MLU = 1 to 1.5), rising to ceiling at MLU 1.5 to 2.

There were no between-languages differences in the percentage of children who produced at least one demonstrative word: determiners/pronouns, χ^2^ (1) = 0.32, *p* = 0.6; locatives, χ^2^ (1) = 1.7, *p* = 0.2; or any demonstrative, χ^2^ (1) = 0.59, *p* = 0.4. Locatives featured more often in children’s vocabulary than determiners/pronouns: in Spanish, χ^2^ (1) = 3.96, *p* = 0.047; and English, χ^2^ (1) = 42.76, *p* < 0.001. In Spanish, this difference was only significant for the youngest age group, 18 to 20 months [χ^2^ (1) = 12.40, *p* < 0.001, Bonferroni adjusted alpha level of 0.017] and at none of the MLU bins. In English it was significant in the two youngest groups [18 to 20 months, χ^2^ (1) = 14.25, *p* < 0.001; 20 to 22 months, χ^2^ (1) = 13.85, *p* < 0.001], and the two lower MLU bins [MLU 1 to 1.5, χ^2^ (1) = 20.42, *p* < 0.001; MLU 1.5 to 2, χ^2^ (1) = 9.27, *p* = 0.002], Bonferroni adjusted alpha levels of 0.017.

#### Most Common Demonstrative Terms in Child Lexicon

**FIGURE 2:**
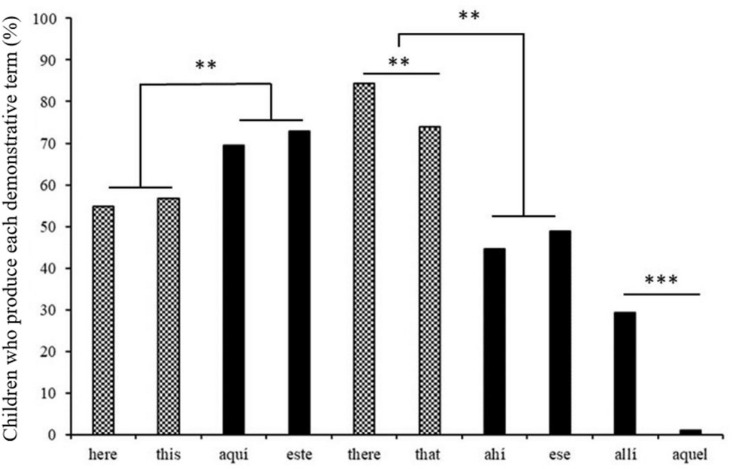
Children who use any demonstrative word in CHILDES corpora, by word (%). *p < 0.05; **p < 0.01; ***p < 0.001.

After finding that demonstratives featured in a similar proportion of Spanish and English transcripts, we tested which demonstrative words occurred in each language, irrespective of how frequently they were used. The percentages of children who used each demonstrative term at least once are displayed in Figure 2. A greater proportion of Spanish children than British children used proximal terms [*este/aquí, this/here*, χ^2^ (1) = 9.5, *p* = 0.002]. Contrarily, English distal terms *that* and *there* appeared in more transcripts than Spanish medial terms *ese* and *ahí* [χ^2^ (1) = 9.78, *p* = 0.002]. Spanish distal terms *aquel* and *allí* were rare:1% of Spanish transcripts featured the demonstrative *aquel* and 28% the locative *allí*.

#### Demonstrative Frequency in Child Speech in Relation to Other Words

Corpora transcripts were processed with the *tidytext* R package (Silge and Robinson, 2016) to extract the most frequent words in both languages. For this descriptive analysis, the *stem* transcript line was used. Some transcripts feature only the *gloss* transcript line. This contains the actual vocalizations of the child, and thus is unsuitable to count frequencies if one wishes to disregard phonetic errors. The *stem* line has the corrected word and the word root in case of verbs. There were 174 transcripts with *stem* line from English children (mean Age = 20 months) and 65 from Spanish children (mean Age = 21 months).

**FIGURE 3:**
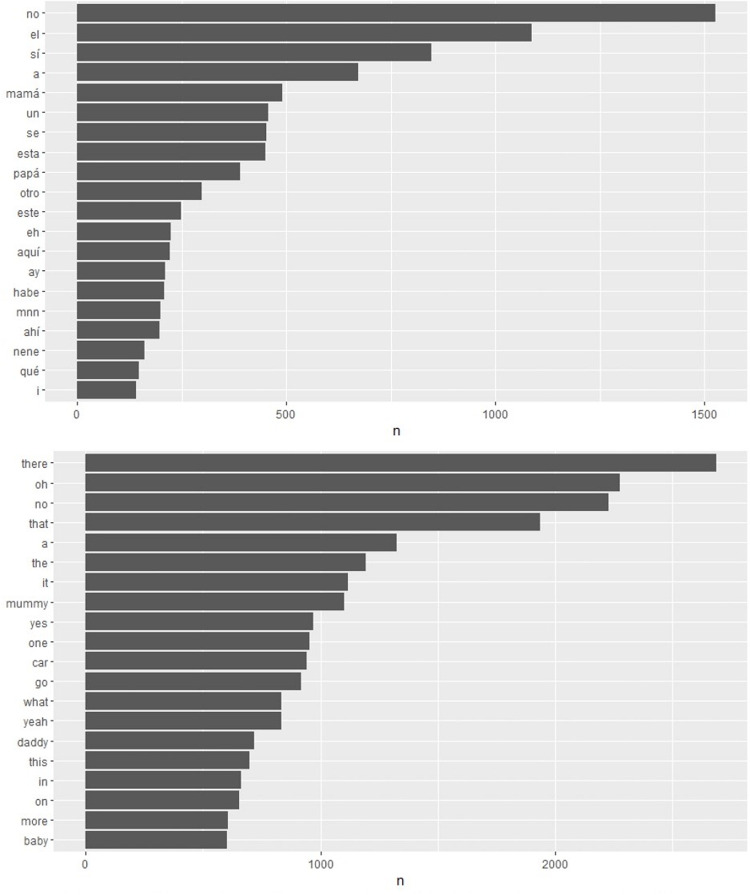
Word frequency of the 20 most frequent words in CHILDES corpora in Spanish, above, and British children, below. Notice in the Spanish plot the 8th word *esta* does not refer to the demonstrative word, but to the root of the verb *estar* (to be).

Word frequencies were computed for all words in all scripts for each language. Figure 3 displays the number of occurrences of the 20 most frequent words for each language. In Spanish, *este* (this), *aquí* (here) and *ahí* (there) were among the 20 most frequent words, in 11th, 13th, and 17th position, respectively. In English, *there, that*, and *this* were among the 20 most frequent words. *There* was the single most frequent word in the corpus, and *that* and *this* occupied 4th and 16th positions, respectively.

#### Demonstrative Frequency in Child and Parent Speech

**FIGURE 4:**
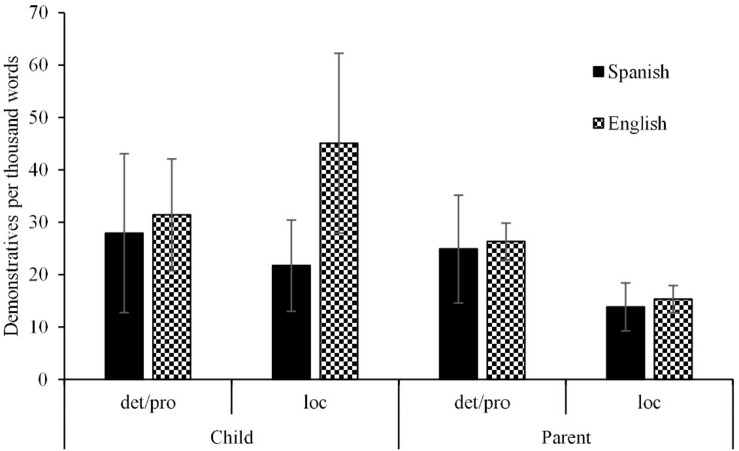
Mean frequency of determiner/pronouns and locatives per thousand words in CHILDES corpora, by language and speaker. Error bars correspond with the 95% confidence interval for mean. Demonstratives were present in all Spanish parents’ transcripts and in 98% of British parents’ transcripts.

The number of demonstratives per thousand words was computed for determiners/pronouns and locatives in both languages and is displayed in Figure 4. In child speech, determiners/pronouns were equally frequent in Spanish and English (28 vs. 31 occurrences per thousand words, Mann-Whitney *U* Test, *Z* = 1.0, *p* = 0.32). However, locatives were much more frequent in English than in Spanish in child speech (45 vs. 22 occurrences per thousand words, *Z* = 3.7, *p* < 0.001). In parent speech, both determiners/pronouns and locatives were slightly more frequent in English than in Spanish (determiners/pronouns, 26 vs 25 occurrences per thousand words, *Z* = 3.6, *p* < 0.001; locatives, 15 vs. 14 occurrences, *Z* = 2.1, *p* = 0.03).

Next, we examined demonstrative frequency across the age and MLU range using correlational analysis^4^. There were positive correlations between MLU and determiner/pronoun frequency in Spanish (*r* = 0.25, *p* = 0.02) and English (*r* = 0.20, *p* = 0.009): determiners/pronouns were more frequent in children with longer MLU. Locative adverbs did not significantly correlate with MLU in Spanish (*r* = 0.17, *p* = 0.11) or English (*r* = −0.10, *p* = 0.19). Age correlated with MLU in English, *r* = 0.40, *p* < 0.001, but not in Spanish, *r* = 0.14, *p* = 0.2. Correlations between demonstrative frequency and age did not approach significance (*r’*s < 0.15, *p’*s > 0.14).

**FIGURE 5:**
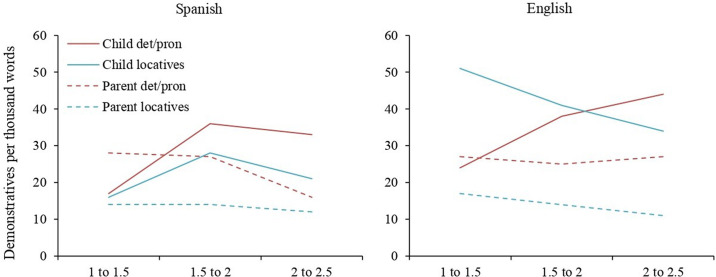
Demonstrative frequency per thousand words for children and parents at each level of MLU in CHILDES corpora.

We also examined possible differences in child-directed speech across development. Parent demonstrative frequency correlated negatively with child MLU: parents used more demonstratives at the early stages of language acquisition and parent usage decreased with child language development: in English, *r* = −0.17, *p* = 0.031, and Spanish, *r* = −0.22, *p* = 0.037. Nevertheless, parents’ and children’s demonstrative frequency correlated positively in English, *r* = 0.41, *p* < 0.001, and Spanish, *r* = 0.277, *p* = 0.008. Changes in frequency of demonstrative words by MLU for children and parents are displayed in Figure 5.

#### Demonstrative Types in Child and Parent Speech

This analysis examined the relationship between the demonstrative words used by each parent-child dyad, particularly whether they tend to use the same demonstrative words during an interaction. A correlational analysis was performed on the frequency of each demonstrative word per thousand words between speakers (parent and child) within transcripts. Results are displayed in Table 3. Parents tended to use the same determiners/pronouns as the children, and rarely used others. This was also the case for distal locatives, but when children used proximal locatives parents were equally likely to use distal or proximal (English), or distal or medial (Spanish).

**TABLE 3:** Within-transcripts correlations between parent and child demonstratives’ frequency per thousand words.

			**Child**					
			**Det/pronoun**			**Locative**		
	**Parent**		**Proximal**	**Medial**	**Distal**	**Proximal**	**Medial**	**Distal**
Demonstrative	Spanish	Proximal	0.21*	−0.00	−0.04	0.26*	0.03	−0.03
		Medial	0.17	0.42**	0.05	0.24*	**0.19**	0.03
		Distal	−0.07	−0.06	**−0.02**	−0.14	0.04	0.40**
	English	Proximal	0.17*	−	0.13	0.22**	−	0.00
		Distal	0.13	−	0.27**	0.23**	−	0.28**

Data from CHILDES. *p < 0.05; **p < 0.01; ***p < 0.001. Bold values indicate the correlation between parent’s and child’s frequency of use of the same word.

#### Conclusions of Study 1 (CHILDES Data)

Analysis of the spontaneous speech of 18 to 24 month old English and Spanish speaking children revealed that demonstratives are used by more than half of children from age 18 months, and at the single-word utterance stage. However, it is not until children are starting to produce two-word utterances that we see demonstratives in nearly all children. There were no significant between-language differences. What CHILDES data do not reveal is the order of acquisition of demonstratives, nor whether they appear among the first 50 words. That will be examined using parental report (CDI) data in Study 2. Findings from the descriptive analysis of CHILDES data on demonstrative use and parental input will be discussed in the General Discussion.

## Study 2 (Based on CDI-Wordbank Data)

Study 2 investigates the acquisition of demonstrative words in English and Spanish using data from parental report. Specifically, we look at when the majority of children use demonstratives with respect to their vocabulary size and age in both languages.

### Method

#### Origin of the Data

Data come from 277 monolingual speakers of European Spanish and 673 of British English, between the age of 18 and 24 months. Sample distribution by age is displayed in Table 4. Data sources: López et al. (2005), Floccia (2017).

**TABLE 4:** Sample size and mean productive vocabulary size and *SD* for each age and language group.

	**Spanish**		**English**	
**Age (months)**	**Sample size**	**Vocabulary Mean (*SD*)**	**Sample size**	**Vocabulary Mean (*SD*)**
18	50	70	118	51
		(79)		(60)
19	27	84	109	82
		(64)		(82)
20	36	117	144	110
		(105)		(93)
21	41	144	75	130
		(105)		(92)
22	38	184	28	151
		(125)		(118)
23	30	230	112	187
		(122)		(121)
24	55	257	87	220
		(161)		(113)
Total sample size	277	No. items:	673	No. items:
		588		418

*Data from CDI Wordbank.*

#### Instrument

The instruments used were the Oxford CDI for British English and the Words and Sentences for European Spanish (Hamilton et al., 2000; López et al., 2005). These questionnaires are not a direct translation of each other, but an adaptation to fit linguistic and cultural differences. Therefore, although they include the same word categories, the Spanish version features more items (588) than the British one (418). The average vocabulary size for each age and language group is displayed in Table 4.

Demonstrative words in the English instrument include *this, that* and *there*, but not *here*, nor the plural forms *these* and *those.* The Spanish questionnaire features all demonstrative words, including gender and number variations (13 items, see Table 1).

#### Data Processing and Analysis

Data were extracted and processed using the *wordbankr* R package (Braginsky, 2018) on 25/11/2019. To make the two languages comparable, in Spanish we worked only with the singular forms of demonstratives^5^. A dummy variable was computed to indicate whether a child produced any demonstrative word, irrespective of the frequency. The percentage of children that produced demonstratives was compared at each Age and MLU level. Age levels were each month from 18 to 24 months. Minimum vocabulary size (CDI score) was binned in groups of 50 words (CDI score of 0 to 50 words, 51 to 100 words, and up to 400). Chi-squared tests on the raw data were used throughout. Two separate analyses were made, one for determiners/pronouns only, and one for all demonstratives including locatives.

### Results

#### Acquisition of Demonstratives by Age in CDI Data

**FIGURE 6:**
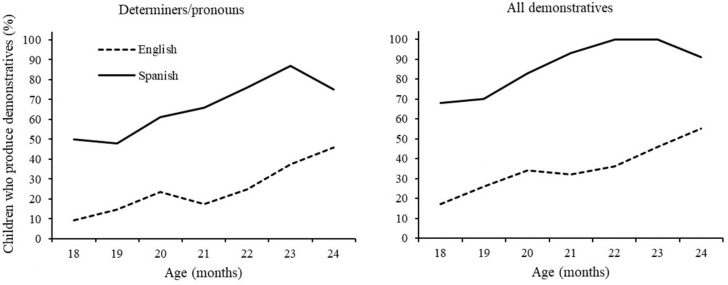
Children who produce any demonstrative word by age and language (%). Data from CDI Wordbank.

Figure 6 displays the percentage of children who used at least one demonstrative word by age and language group. From 21 months onward, more than half the Spanish children used at least one determiner/pronoun (*este, ese* and/or *aquel*). Including locatives, 68% of Spanish children produced at least one demonstrative word from 18 months, and approached 100% at 22 months. In contrast, only 9% of British children produced at least one determiner/pronoun word by 18 months, 17% when including locatives. At 24 months, less than 50% of English speakers produced determiner/pronouns, and 55% when including locatives. At any age point, a greater number of Spanish children compared to British children produced at least one demonstrative, whether or not locatives were included in the analysis [all χ^2^s (1) > 10, *p’*s < 0.001, Bonferroni adjusted alpha level of 0.007].

#### Acquisition of Demonstratives by Vocabulary Size in CDI Data

**FIGURE 7:**
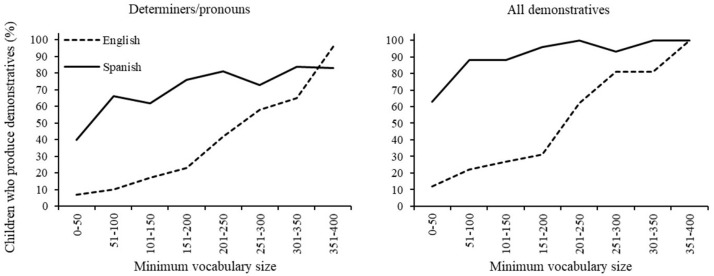
Children who produce demonstrative words by vocabulary size (%). Data from CDI Wordbank.

Figure 7 displays the percentage of children who used demonstratives by minimum vocabulary size (CDI score) for each language. Less than half of the English speakers produced determiners/pronouns below a vocabulary of 300 words. Including locatives, more than half of the children produced at least one demonstrative from 200 words on, and reached ceiling after 350 words. For the Spanish sample, more than half of children produced determiners/pronouns from a vocabulary of 50 words on, and when including locatives, from 0 to 50 words, reaching ceiling at a vocabulary of 150-200 words. More Spanish children than British children produced demonstratives up until a vocabulary of 250 words, either considering determiners/pronouns alone or with locatives [all χ^2^s (1) > 10, *p’*s < 0.001, Bonferroni adjusted alpha level of 0.006]. There were no significant between-language differences thereafter [all χ^2^s (1) > 3, *p’*s > 0.1].

#### Conclusions of Study 2 (CDI-Wordbank Data)

Data from parental report reveal important crosslinguistic differences. The majority of Spanish speakers use at least one demonstrative from 18 months and among their first 50 words if locatives are included, whereas English speakers do not use demonstratives up until age two and a vocabulary size of 200 words, and even later if considering determiners/pronouns only. It was expected that fewer children would use demonstratives in CDI data compared to CHILDES data. However, the striking crosslinguistic differences solely in CDI data suggest possible sampling differences.

#### Demonstrative Production and Parental Education in the Spanish Sample

In the Spanish CDI sample, high education families were over-represented, with 77% of parents having college and graduate education. Maternal education is not reported in the British data, although it is presumably lower, since authors state that their sample SES was representative of the British population (sample composite or SES measurement were not reported in detail; Hamilton et al., 2000). Thus, our hypothesis is that the lower report of demonstrative use in British sample is due to the higher proportion of parents with low education, and the associated bias of underestimating children’s knowledge of function words (Fenson et al., 1994). This was tested by analyzing the differences in report of demonstrative words between high education level (college and University, *n* = 222) and low education level parents (primary and secondary school, *n* = 52) in the Spanish sample (missing cases, *n* = 3). The mean age of children of both groups did not differ significantly (Mann-Whitney U, *Z* = −0.38, *p* = 0.7), nor the total CDI score (Mann-Whitney U, *Z* = −0.65, *p* = 0.5). More parents with higher education reported that their children used demonstratives, 88% vs 77%, χ^2^(1) = 4.56, *p* = 0.03. This supports the hypothesis that parental education might play a role in their accuracy in reporting demonstrative production. However, only 34% of British parents from our data reported demonstrative use, thus sampling issues cannot fully account for the cross-linguistic differences in Study 2.

## General Discussion

This work aimed to describe the acquisition and use of demonstrative words in infants and possible cross-linguistic differences. In Study 1, we analyzed corpus data, that allow measurement of mean length of utterance (MLU), word frequency and parent input. In Study 2, we looked at data from parental report, that feature a measure of vocabulary size and a large sample size. Results will help understand the role of demonstrative words in deictic communication and language acquisition in infancy. They are also interesting from a methodological point of view, contributing to assessing the suitability and validity of parental report and corpus analysis in the study of function words.

First, we asked whether demonstratives appear among children’s first 50 words and at the earliest stages of language development (18 months). Results on age of acquisition differ between measures: according to the CDI results (Study 2), only around half of the English speakers use demonstratives by 24 months, whereas nearly all Spanish speakers used at least one demonstrative by the age of 22 months. In contrast, corpus data (Study 1) indicated that the majority of children of both languages produced at least one demonstrative word from 18 months and all of them did at 24 months. Data from CHILDES indicates that the majority of children from both languages use demonstratives from MLU 1 to 1.5, and reach ceiling with an MLU of 1.5 to 2. Data from the CDI showed at what point in vocabulary acquisition demonstratives appear. The majority of Spanish speakers have a demonstrative among their first 50 words (after the 50th word if considering determiners/pronouns only), reaching ceiling after the 150th word. In contrast, the majority of English speakers do not use demonstratives before their 200th word, reaching ceiling only after their 350th word. This reflects a great discrepancy between CDI and CHILDES data, and it is unclear which one of these sources reflects a more accurate estimation. Nevertheless, we can confidently say that demonstratives do not typically appear before the 50th word, and they are more frequent in two-word utterances. We cannot make any firm statement about possible cross-linguistic differences because the results we obtained were very different between the two sources. We will discuss the possible methodological and sampling sources of discrepancies.

It was expected that the CDI data would underestimate demonstrative production with respect to corpus data (Salerni et al., 2007); however, CDI data also show striking differences between languages, while the corpus data do not. We suggested that differences might be due to sample SES disparity between languages and measures. This bias could have affected the results at two levels: first, because children of parents with higher education levels have an advantage for language development (Hoff, 2006); and second, because parents of low educational level may underestimate children’s knowledge of function words in language inventories (Fenson et al., 1994). In contrast to the CDI data, the CHILDES sample for English may have an overrepresentation of higher SES families: one of the two largest corpora that compose the English corpus (Manchester corpus) is formed of middle-class families, while the other (Wells) has a representative sample extracted from the birth censuses. Thus, the average SES level in the British sample might be higher in CHILDES than in CDI data. Comparisons between high and low education parents in the Spanish sample support the hypothesis that low educational level parents might underestimate their children’s use of demonstratives, but it is unlikely that it can fully explain the magnitude of the differences between languages in CDI data. One possibility is that language-specific factors, such as phonetics, might pose a disadvantage for the identification of demonstratives in English. Having listened to several CHILDES transcripts, our subjective impression is that young infant’s verbalizations of *there* and *that* were often hard to distinguish from babbling, whereas the Spanish words *esto* or *aquí* were easier to recognize, perhaps because they are disyllabic words.

As argued in the introduction, neither checklist nor observational methods alone are ideal for estimating the proportion of particular word types in children’s early vocabulary (Pine et al., 1996). However, combining both methods did not offer conclusive results either, because it is unclear whether the disparity between the two studies is due to methodological or sampling differences. We encourage researchers to take into consideration demographic variables in studies of this kind, while further research that will apply both methods to the same participants is needed to evaluate its impact in the results.

The second aim of this work was to describe the use of demonstratives in child spontaneous speech (Study 1). The analysis of CHILDES data revealed no significant differences between languages in the acquisition of demonstratives with respect to age and MLU. However, it did show that proximal demonstratives appear more often in Spanish and distal demonstratives in English, both in terms of frequency of use and of percentage of children using them at least once. Thus, whereas the use of demonstratives by infants is not a language-specific communicative tool, the preferred demonstrative term varies across languages.

One striking finding is that locatives and determiners/pronouns do not seem to have the same function in language development. Locatives appear earlier and are more frequent, particularly in English and in earlier stages. They are less complex than determiners/pronouns, which are more frequent in children with higher MLU. The most salient difference between languages in children transcripts is in the locative *there/ahí*. In English, it was the most frequent word in children’s lexicons, and its frequency was particularly high in the youngest children. In contrast, the Spanish equivalent *ahí* (and the proximal *aquí*) was no more frequent than the determiner/pronouns. Our hypothesis is that *there* in English (unlike locative adverbs in Spanish) functions as a fixed expression instead of a deictic term, or as a verbalization linked to a particular action. This was the case for the children studied by Harris et al. (1988) and Barrett et al. (1991), who found that children acquired *there* among the first 10 words, but they used it in a very specific context: for example, one participant would only use it with the action of handing a toy. This use might be a precursor of the acquisition of deictic words (i.e., of generalizing *there* to indicate location). However, the analysis of transcripts provides limited context, particularly those of infants in the single-word stage, and thus makes it difficult to assess when children use demonstratives in a ritualistic way or as a deictic communication tool. Future research in the development of deictic communication might take this into consideration, and perhaps analyze separately determiners/pronouns and locatives.

Another interesting difference between the two languages is in the frequency of demonstratives: in English, two demonstratives, *there* and *that*, were among the five most frequent words of child’s lexicon, whereas in Spanish the most frequent demonstratives, the proximal terms *este* and *aquí*, are the 11th and 13th most frequent words. Demonstrative words were also very common in parent speech, although parents used fewer demonstratives than children per thousand words, presumably due to their larger vocabulary.

The analysis of spontaneous speech also allowed description of parent use of demonstratives. Data revealed that parents use more demonstratives in children’s earlier stages of language development, as indicated by a negative correlation between parents’ frequency of demonstratives and children’s MLU. This might indicate that parents move on to use words that are more complex than demonstratives at the moment in their child’s language development when they are acquiring new words at a fast rate.

Interestingly, the frequency of use of each demonstrative term correlated between parent and child. This has potentially interesting implications for later development of spatial demonstratives to convey distance and semantic information. That parent and child are using the same demonstrative word in a given speech suggests that children are not switching the demonstrative term, as happens in adult speech: frequently in an interaction with objects, the speakers view them from opposite sides and therefore use opposed demonstratives (the speaker may use *this* for an object closer to them, whereas the conversational partner refers to the same object with *that*). Our hypothesis is that parents repeat the demonstrative that the child uses in order to reinforce their word learning, while the spatial content of demonstratives (close or far) is not relevant at this stage. Taumoepeau and Ruffman (2008) have demonstrated that mothers are sensitive to what their child can and cannot understand in this age range; when talking about mental states, the speech parents use is only slightly more complex than their child’s current level and within their zone of proximal development (Vygotsky, 1980), plausibly in order to aid their learning. This would predict that parents use demonstratives without considering their spatial dimension or deliberately adopt their child’s perspective when the distance contrasts are too complex for the child’s current level. One example of such behavior might be in the following script (Anne, 1;11, free play with mother).

Child: What [is] baby doing?
Mother: Which baby?
Child: This baby.
Mother: This one?
Child: Yeah
Mother: Oh dear that baby’s fallen out of the pram.

In this example, the child uses the proximal demonstrative, then the mother repeats it, but her next sentence features the distal demonstrative for the same referent. The child, mother, and the referent (the baby doll) do not apparently change location during the exchange, so the mother’s appropriate demonstrative would have been *that*. However, the mother first repeated the child’s demonstrative as a reinforcement. Here is another example, in Spanish (Mendía, 1;08, free play with mother, includes video):

Child and mother are playing on the floor. Child turns around and refers to a game that is located slightly further, indicating that he would like to play with it some more. The child uses the proximal demonstrative and the mother uses it too.
Child: éte [: éste]. - *This.*
Child: má [: más]. - *More.*
Mother: muy bien (.) ¿más? - *Very well. More?*
Child: má [: más]. - *More*
Mother: ¿éste? - *This one?*
Mother: ¿hacemos éste otra vez? - *Do we do this one again?*
Child: títo [/] [?].
Mother: ¿éste otra vez? - *This one again?*

This hypothesis, however, should be taken with caution, since there are frequent examples where it does not occur. There are also numerous events in which it cannot be assessed because only parent or child use demonstratives. Parents’ use of demonstratives according to the child’s perspective might be limited to a specific developmental stage. Further research could investigate parent-child synchrony of demonstratives in video-recorded interactions, to see at what stage in development parents take their children’s perspective with demonstrative words and how it influences their subsequent acquisition of the spatial contrast.

Results from the CHILDES corpora are to be interpreted with caution because of the small sample size in Spanish (seven children). Individual differences and preferences might have been overrepresented in our results. The CHILDES database would benefit from more contributions of early speech in languages other than English. Particularly, parent-child interactions in video format would be a valuable addition to the study of deictic communication in infancy.

## Conclusion

We studied the acquisition and frequency of demonstrative words in English and Spanish using transcripts of spontaneous speech and parental report data. Results indicate that demonstratives do not typically appear before the 50th word and are more frequent at the two-word-utterance stage than at the onset of productive language. This work challenges previous claims about the acquisition of demonstratives (Clark, 1978). In line with other studies that have looked at deictic communication in infants (Capirci et al., 1996; Rodrigo et al., 2004), we conclude that demonstratives may not be the most frequent means of early verbal deixis; other words or verbalizations may take that function earlier in development, whereas demonstratives become more frequent in more elaborate utterances later on. Our work is limited to two languages and shows important discrepancies between measures; nevertheless, it might encourage researchers to pay closer attention to other word types or vocalizations when studying verbal deixis in early language development.

From a methodological point of view, comparing parental report and spontaneous speech data in the study of function words has highlighted the potential limitations of both measures. Further research needs to examine the suitability, limitations, or improvement of both methods for the study of function words in child speech.

## Data Availability Statement

The datasets generated for this study are available on request to the corresponding author.

## Author Contributions

PG-P contributed with the conceptualization, methodology, investigation, data analysis, visualizations, theoretical framework, results interpretation, and writing. PG-F contributed with the conceptualization, supervision, theoretical framework, results interpretation, review, and editing. MD contributed with the theoretical framework, results interpretation, review, and editing. All authors contributed to the article and approved the submitted version.

## Conflict of Interest

The authors declare that the research was conducted in the absence of any commercial or financial relationships that could be construed as a potential conflict of interest.

## Footnotes

1 http://wordbank.stanford.edu

2 An alternative spelling of demonstratives in Spanish, now obsolete, features a written accent on the demonstrative pronouns (éste, ése…) to differentiate them from the determiners. *Childesr* word retrieval is sensitive to written accents, and we included both spelling forms in our search. The sensitivity to written accents allowed distinction of the verb form *está* (is) from the proximal, female demonstrative *esta*/*ésta*.

3 We considered filtering out the non-demonstrative uses of these words using the MOR line of the transcripts, that specifies the word class of each word. However, after analyzing several transcripts, we found this categorization to be unreliable for demonstratives. Nevertheless, although the results might overestimate demonstrative use in English for parents, we do not consider this a serious concern for child data.

4 Due to the number of outliers in the sample, bootstrap based on 1000 bootstrap samples was calculated. In none of the significant correlations did the 95% bootstrap confidence interval contain zero; therefore, we can be confident of the correlations’ significance.

5 None of the children produced only plural forms of demonstratives; plural forms in Spanish were always acquired after the singular forms. Therefore, this selection had no effect on the findings.
